# Analysis of Incident Reports of a Dental University Hospital

**DOI:** 10.3390/ijerph18168350

**Published:** 2021-08-06

**Authors:** Yasuyuki Kimura, Ken-ichi Tonami, Akira Toyofuku, Hiroshi Nitta

**Affiliations:** 1 Oral Diagnosis and General Dentistry, Dental Hospital, Tokyo Medical and Dental University, Tokyo 113-8549, Japan; ken1.gend@tmd.ac.jp (K.-i.T.); nitta.behd@tmd.ac.jp (H.N.); 2 Medical Safety Management Office, Dental Hospital, Tokyo Medical and Dental University, Tokyo 113-8549, Japan; toyoompm@tmd.ac.jp; 3 Comprehensive Patient Care Psychosomatic Dentistry, Graduate School of Medical and Dental Science, Tokyo Medical and Dental University, Tokyo 113-8549, Japan

**Keywords:** dentistry, medical safety, incident reporting

## Abstract

Incident reports are important for improving the quality and safety of medical care. Healthcare workers with less than one year of work experience have been reported to cause the most incidents, and the most common incident is “drug-related”. However, few studies have comprehensively analyzed incidents in dentistry, and the characteristics of dental incidents have not been understood. In this study, to understand the characteristics of dental incidents, we comprehensively analyzed 1291 incident reports submitted to the Tokyo Medical and Dental University Dental Hospital from April 2014 to March 2019. As a result, dental outpatient and dental wards had different types of incidents. In outpatient wards, incidents included many dentistry-specific incidents related to “procedures”. Among them, “poor physical condition of the patient during dental treatment” was the most common incident. In contrast, the most common incident from subjects with less than one year of work experience was “damage to soft tissues around the teeth”. Thus, to improve the quality and safety in dentistry, it is was considered necessary to analyze and understand the characteristics of dentistry-specific incidents and to take appropriate measures and educate dental professionals.

## 1. Introduction

In 1999, “To Err is Human” [1], reported by the US Institute of Medicine’s (IOM), revealed the current number of deaths due to medical errors and stated that this number can be reduced by taking appropriate measures. This has increased the world’s awareness of medical safety. Similarly due to multiple serious medical accidents in 1999 in Japan, social interest in medical safety has increased, and the country began to work on improving the quality of medical care. At each medical institution, the establishment of a system to prevent serious medical accidents was promoted. In 2004, the World Health Organization (WHO) launched the World Alliance for Patient Safety [2]. Reducing medical errors is now a major international concern.

In hospitals, incident reporting systems are used to collect information about incidents that have or are likely to affect patients, such as patient injuries, equipment failures, and near misses. Incident reports are important for improving the quality and safety of medical care. Incident reporting systems have been introduced in many Japanese hospitals [3,4]. To promote medical safety countermeasures in dentistry, it is necessary to collect and analyze incidents occurring in dental medical institutions. However, at present, few reports have comprehensively analyzed incidents in dentistry, and the characteristics of these dental incidents have not been understood. Therefore, current dental safety only depends on the experience of each dentist, and systematic dental safety education has not been established.

Healthcare professionals with less than one year of work experience tend to cause the most incidents, [5,6] with “drug-related” incidents being the most common [6,7,8]. However, whether there is a similar tendency in dentistry is uncertain. The purpose of this study is to analyze the characteristics of dental incidents. For that, in this study, we comprehensively analyzed incident reports submitted to our dental university hospital from the past five years, and assessed differences due to years of occupational experience.

## 2. Materials and Methods

### 2.1. Subjects

We assessed 1291 incident reports submitted to the Dental Hospital, Tokyo Medical, and Dental University, from 2014 of Japanese fiscal year (hereafter, FY) to FY2019. Approximately 1600 to 1800 patients visit the dental hospital each day. All the incidents happened in the management area in the dental hospital. An existing incident collection system was used for data collection. In this system, hospital staff voluntarily describe incidents encountered in their working hours. Convenience sampling was employed; that is, all the data in the incident collection system were used for analysis, and any randomization was not conducted. The subject of this survey is completely anonymous, and there is no personally identifiable information. Therefore, the University Ethics Review Committee had stated that ethics review was not required.

### 2.2. Methods

The subjects were analyzed, focusing on the reporter, content, location, cause, and degree of impact to the patient. In this study, “incidents” were defined as the occurrence of actions or situations that deviate from normal during dental treatment. This includes incidents with and without injury to the patient, and those with and without the negligence of the dental healthcare workers.

#### 2.2.1. Classification by Occupation and Department of Reporter

The subjects were classified according to their occupation. The report rate of dentists was aggregated every fiscal year, and the subjects were classified according to the department to which the reporter belongs.

#### 2.2.2. Classification by the Work Experience

The subjects were classified according to their years of work experience, into four groups, “0–9 years”, “10–19 years”, “19–29 years”, and “30 years or more” and were statistically compared. In addition, the “0–9 years” group was subdivided into 0 to 9 years and was statistically compared.

#### 2.2.3. Classification by the Contents of the Incidents

The subjects were classified according to the content of the report into the following eight groups: “procedures”, “medical equipment”, “drugs”, “examinations”, “turning over, falling down”, “blood transfusions”, “drains”, and “others”. Each group was aggregated by location: “dental ward/central operating room” and “outpatient”. Differences between “dental ward/central operating room” and “outpatient” regarding each contents of incident reports were statistically analyzed using the Chi-square test and Fisher’s exact test.

In addition, “procedures” were subdivided into 10 items with reference to the classification of the incident report system at our hospital: “poor physical condition of the patient during dental treatment”, “damage to soft tissues around teeth”, “accidental ingestion/aspiration”, “misidentification of patients”, “injury by sharp instruments”, “contamination of clothes or belongings”, “loss of instruments”, “damage to teeth”, “mistakes in parts”, “anesthesia”, and “other treatments”. These incidents were compared between subjects with less than one year of work experience and those with more than one year of work experience. Regarding each contents of incident reports, differences between dental workers of less than 1 year of work experience and those of more than 1 year were statistically analyzed using the Chi-square test and Fisher’s exact test.

#### 2.2.4. Classification by the Causes of the Incidents

Based on the classification by Watanabe et al. [9], the subjects were classified according to the cause of incidents into the following eight groups: “confirmation” (lack of confirmation, lack of observation, etc.), “knowledge/experience” (lack of knowledge, lack of technology, unfamiliarity with instruments, etc.), “cognition” (belief, forgetfulness, etc.), “cooperation/information transmission” (insufficient explanation, communication, etc.), “working status” (I was in a hurry, etc.), “judgment” (inappropriate judgment, etc.), “patient” (patient and family factors), and “other” (impossible, depending on medical equipment).

#### 2.2.5. Statistical Analysis

Statistical analysis software SPSS ver. 27 (IBM, New York, NY, USA) was used for all statistical analyses. The level of significance was 5%.

## 3. Results

**Figure 1:**
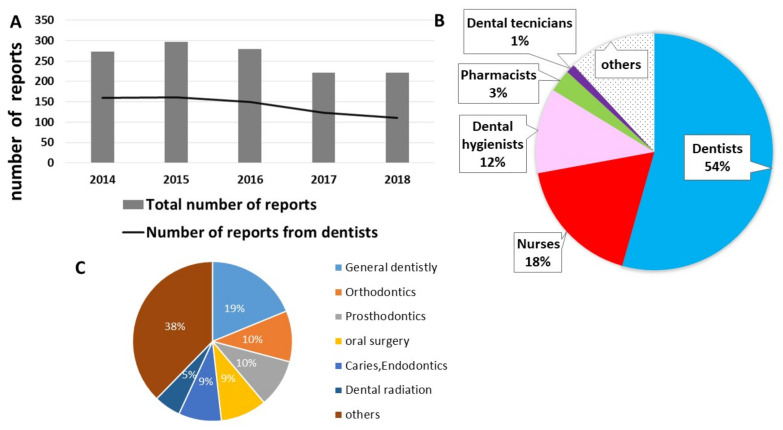
(**A**) The total number of incident reports, and the number of incident reports by dentists. (**B**) Rate of the occupations submitted incident reports. (**C**) Rate of the departments submitted incident reports.

Approximately 250 incidents were reported each year during the five-year survey. Among them, reports from dentists remained at approximately 50% every year (Figure 1A). The occupations of the incident reporters were dentists (54%), nurses (18%), and dental hygienists (12%) (Figure 1B). Focusing on the department to which the reporter belongs, the number of incident reports from general dentistry (most are residents and students) was the greatest (Figure 1C).

**Figure 2:**
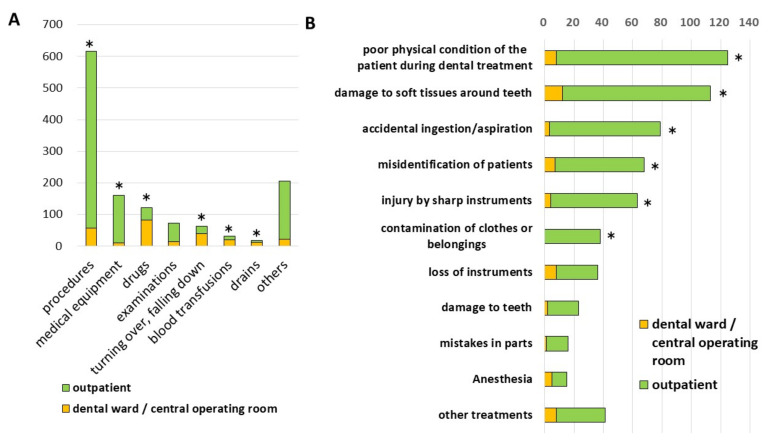
(**A**) The number of incident reports from different location. (* *p* < 0.05) (**B**) The classification of the incident reports related to the procedure from different location.

We analyzed the contents of the incident reports and found that “procedures” was the most common among all reports. In terms of the location, the most common incident at the “dental ward/central operating room” was “drug-related”, while that at the “outpatient ward” was “procedures” (Figure 2A). In the dental ward/central operating room, the numbers of incident reports related to “drugs”, “turning over, falling down”, “blood transfusions”, and “drains” were significantly higher than those in outpatient wards (*p* < 0.05). On the other hand, in outpatient wards, the numbers of incident reports related to “procedures” and “medical equipment”, were significantly higher than those in the “dental ward/central operating room” (*p* < 0.05). We further classified “procedures” into 10 groups. We found that “poor physical condition of the patient during dental treatment” was the most common cause, followed by “damage to soft tissues around teeth” and “accidental ingestion/aspiration” (Figure 2B). The numbers of incident reports related to “poor physical condition of the patient during dental treatment”, “damage to soft tissues around teeth”, “accidental ingestion/aspiration”, “misidentification of patients”, “injury by sharp instruments”, and “contamination of clothes or belongings” were significantly greater in outpatient wards than those in dental ward/central operating rooms, but there was no significant difference in “loss of instruments”, “damage to teeth”, “mistakes in parts”, and “anesthesia”.

**Figure 3:**
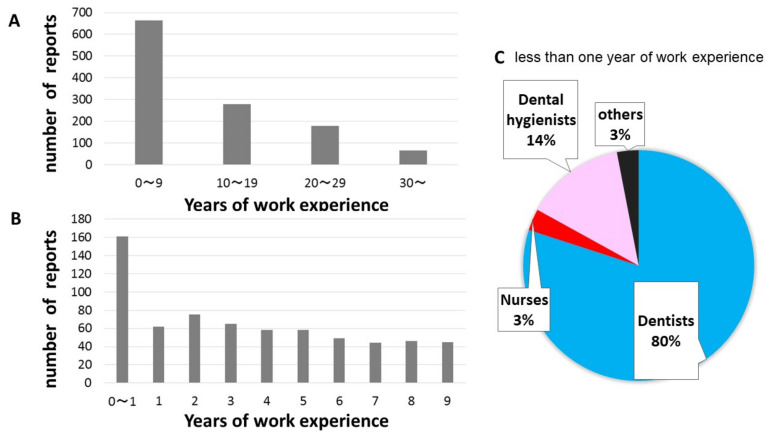
(**A**) Relationship between years of work experience every 10 years and number of incident reports. (**B**) Comparison of the number of reports from subjects with 0 to 9 years of work experience. (**C**) Report rate by occupation among dental care workers with less than one year of work experience.

In terms of work experience, the number of incident reports was higher in the ”0–9 years” group than in the other groups, and the number of incident reports tended to decrease as the years of work experience increased (Figure 3A). When comparing subjects in the “0 to 9 years” group, subjects with less than one year of work experience had the highest number of incident reports (Figure 3B). In terms of subjects with less than one year of work experience, approximately 80% of the reports were from dentists (Figure 3C).

**Figure 4:**
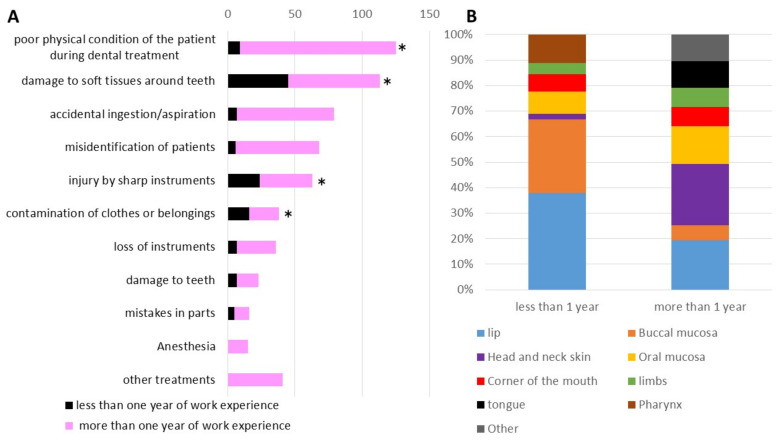
(**A**) Comparison of incidents related to procedure reported by dental care workers with less or more than one year of work experience. (* *p* < 0.05) (**B**) Comparison of injury site of “damage to soft tissues around teeth with less or more than one year of work experience”.

Among the incidents related to “procedures” from subjects with less than one year of work experience, “damage to soft tissues around teeth” was the most common, which was followed by “injury by sharp instruments” and “contamination of clothes or belongings” (Figure 4A). Regarding “poor physical condition of the patient during dental treatment”, “damage to soft tissues around teeth”, “injury by sharp instruments”, and “contamination of clothes or belongings”, the numbers of incident reports from subjects with less than one year of work experience were significantly higher than those with more than 1 year. Regarding the injury site, the lip and buccal mucosa were more common among subjects with less than one year of work experience (Figure 4B). In addition, injury to the buccal mucosa tended to occur more frequently in subjects with less than one year of work experience. In terms of injury by sharp instruments, scaler tips caused 30.4%, rotary cutting tools caused 13.0%, injection needles caused 8.6%, and scalpels caused 8.6% of injuries.

**Figure 5:**
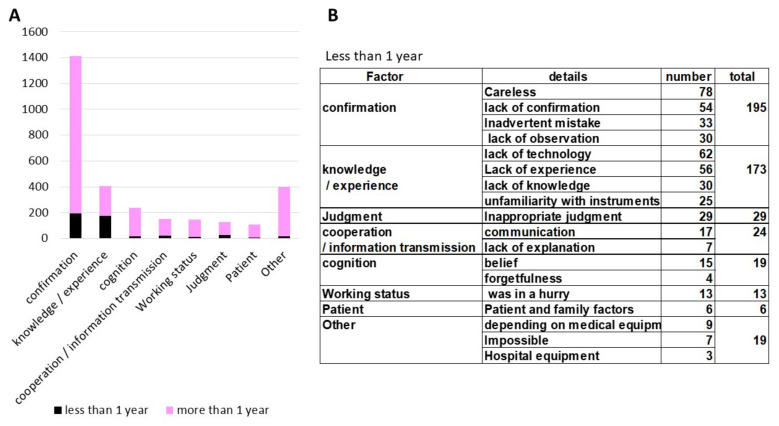
(**A**) Comparison of factors of incidents reported by dental care workers with less and more than one year of work experience. (**B**) The cause of the incidents in dental care workers with less than one year of work experience.

The most common factor of incidents among dental care workers with one year or more of work experience was lack of “confirmation” (48%). For dental care workers with less than one year of work experience, two items, “confirmation” (41%) and “knowledge/experience” (36%) (Figure 5) were the most common causes of incidents.

## 4. Discussion

We comprehensively analyzed incident reports submitted to our dental university hospital from the past five years. Incident reports from nurses tend to be high in many hospitals [6,10]; however, in our hospital, incident reports from dentists were the highest, at approximately 50%. According to a report by Miwa et al. [10], in 2001, the percentage of incident reports from nurses was 66% and that from dentists was 32%, indicating that the percentage of reports from dentists is increasing. We believe that this increase in incident reports by dentists may be because our hospital currently has more dentists (356) than nurses (56), and the risk management working group was established at our hospital in 2004, which raised the dentist’s awareness of safety. There have been differences in reporting depending on the time of day [11], but no significant difference was found at our hospital.

This study found that dental outpatient and dental wards have different types of incidents. In the dental ward, there were many cases of “drug-related” incidents, which was similar to that reported in the medical department [6,7,8]. In the dental outpatient department, most incidents were related to “procedures”, namely “poor physical condition of the patient during dental treatment”, “damage to soft tissues around teeth”, and “accidental ingestion/aspiration”, respectively, all of which were incidents specific to dentistry. The reason for the high number of patients with a poor physical condition during dental treatment may be the high proportion of patients aged 60 years and over visiting our hospital, as well as since Japan is entering a super-aging society [12]. Furthermore, the prevalence of hypertension, kidney disease, and cerebrovascular disease is high, even in younger patients at our hospital [13]. A lot of the “poor physical condition of the patient during dental treatment” reported in this study were after dental anesthesia. One of the leading causes of death reported in dentistry was anesthesia/medication-related [14]. Local anesthetics commonly used in dentistry contain epinephrine to constrict blood vessels and reduce bleeding; thus, they have been reported to increase blood pressure as a side effect [15]. Hypertensive patients are five times more sensitive to the effects of epinephrine [16]. According to the guidelines, referral to a doctor is prioritized if blood pressure is ≥180/110 mmHg except in an emergency [17,18]. To reduce the “poor physical condition of patients during dental treatment”, it is important for the dental care worker to probe for their medical history before dental treatment at the first visit and check patient’s blood pressure before local anesthetics and surgical invasion. To improve the quality of dental care, it is necessary to analyze and understand the characteristics of incidents unique to dentistry in the outpatient department and to take appropriate measures and education for dental professionals.

Most dental incident reporters had less than one year of work experience, which is similar to the analysis at the Medical University Hospital [6]. Regarding incidents reported by subjects with less than one year of occupational experience, “damage to soft tissues around teeth” was the most common. Damage of lip and buccal mucosa were more common in subjects with less than one year of experience when compared with the overall tendency. Risk management education for workers with less than one year of experience may effectively prevent tissue damage and reduce dental incidents. Regarding the second most common cause, “injury by sharp instruments”, injury by the scaler tip was more common than that by an injection needle or scalpel blade, because the scaler tip has no blade. We believe that this may be because the instruments were not handled carefully. Therefore, it is necessary to educate dental workers with less than one year of work experience that even the tip of a scaler can injure patients if mishandled. The third most common cause, “contamination of clothes and belongings”, is also an incident report specific to dentistry. In dental treatment, colored liquids, such as caries detection liquid [19] and plaque dyeing agent [20], are frequently washed in the oral cavity. In addition, the sodium hypochlorite aqueous solution used for dental root canal treatment has a decolorizing effect. Due to many incident reports on these, it was considered necessary to educate dentists with less than one year of work experience on these effects, incident examples, and recurrence prevention measures.

We found that the most common causes of incidents considered by the parties themselves are related to “confirmation”, such as lack of confirmation. On the other hand, in terms of subjects with less than one year of work experience, we found that the number of incidents related to “knowledge/experience” was similar to those related to “confirmation.” It is important for safety management to create a system to reduce these issues. In a survey, US surgical residents said that they lacked knowledge about medical malpractice [21]. Medical safety education is considered effective in avoiding a lack of knowledge. However, it was reported that it is difficult to gather staff for safety education and training due to many shift work routines [22,23]. Education through e-learning might help improve knowledge and skills by repeatedly learning according to each person’s schedule and understanding level using a personal computer or simulator equipment [24]. Simulation education using VR (virtual reality) and AR (augmented reality) for acquiring dental treatment techniques might be developed. In recent years, the literature on e-learning related to medical safety has been increasing [25]. It is necessary to develop e-learning strategies in dental medical safety.

To improve the quality and safety of dental treatment, it is important to share the incident analysis results within the organization and go through the PDCA (Plan Do Check Action) cycle [26]. To that end, it is important to efficiently collect a large number of incident reports. However, all the information about the incidents in our hospital might not come to us. This is because it has been identified that the awareness and attitude of students and residents regarding incident reports is an issue [27]. In addition, although general dental clinics play a central role in Japanese dentistry, it is difficult to share an incident report because few general dental clinics have introduced a reporting system. Therefore, building a system that makes it easy for small general dental clinics to report and share daily incidents is very useful to strengthen dental safety measures. In addition, to go through the PDCA cycle, it is important to provide appropriate feedback about incidents to dental workers. However, regular meetings with other hospitals on medical safety often raise the issue that feedback on medical safety is not communicated to all medical professionals in the hospital, especially young medical professionals. This issue is considered for the future.

This study has potential limitations. The incident reports are voluntarily submitted. Thus, the data used in this study might not cover all the incidents that happened during the survey period in the hospital. Additionally, all incidents reported and submitted were used for the analyses; that is, not random sampling but convenience sampling was employed as the sampling method. As a result of such limitation in sampling, comparisons among departments were not conducted. All the incident reports were comprehensively analyzed to describe the present state of the dental hospital as a whole. The obtained new findings are vulnerable because no study has been reported the analyses of incidents at such a big dental hospital. Further examination should be done to clarify the details of the incident in a dental hospital using more sophisticated sampling procedures.

Finally, during the second half of FY2019 and FY2020, the world was affected by the new coronavirus, SARS-CoV-2, which had a great impact on medical care [28]. Our hospital was partially closed, and the number and content of incident reports had changed. In addition, since the incident reporting system at our hospital was changed from FY2019, it would be difficult to compare incidents under the same conditions; therefore, these data were not included. The COVID-19 pandemic has forced dental care workers to change infection control strategies [29,30]. There has been a reported change of outpatient oral surgery during the COVID-19 pandemic [31]. It was reported that extraoral dental vacuums are an effective method of reducing droplet spatter during operative dental procedures and can help reduce the risk of COVID-19 spread during dental procedures [32]. At our hospital, dental workers are obliged to wear personal protective equipment (PPE) during dental treatment to prevent the new coronavirus SARS-CoV-2 infection. With the introduction of PPE and extraoral dental vacuums, a new type of incident emerged that was not seen before the coronavirus pandemic. In the future, we plan to keep follow-up reports on changes caused by the above-mentioned factors. In this way, it was found that the content of the incident reports will change depending on the social situation and changes in medical equipment. We thought that the analysis of incidents in dentistry such as this study should be done regularly.

## 5. Conclusions

We comprehensively analyzed 5-year incident reports submitted to our dental university hospital. Dental outpatient and dental wards had different types of incidents. There were many “drug-related” incidents reports in the dental ward; conversely, in the dental outpatient department, incident reports in dentistry included many dentistry-specific incidents related to “procedures” such as “poor physical condition of the patient during dental treatment”. Most dental incident reporters had less than one year of work experience. The tendency of incidents to occur differed depending on the years of work experience. It was considered necessary to educate dentists with less than one year of work experience on incident examples and recurrence prevention measures.

## 6. Patents

This research was supported by JSPS Grant-in-Aid for Scientific Research (20K18825).

## Data Availability

The data presented in this study are available from the corresponding author, Y.K., upon reasonable request.
